# USR-VS: a web server for large-scale prospective virtual screening using ultrafast shape recognition techniques

**DOI:** 10.1093/nar/gkw320

**Published:** 2016-04-22

**Authors:** Hongjian Li, Kwong-S. Leung, Man-H. Wong, Pedro J. Ballester

**Affiliations:** 1 Institute of Future Cities, Chinese University of Hong Kong, Hong Kong; 2 Department of Computer Science and Engineering, Chinese University of Hong Kong, Sha Tin, New Territories, Hong Kong; 3 Cancer Research Center of Marseille, INSERM U1068, 13009-Marseille, France

## Abstract

Ligand-based Virtual Screening (VS) methods aim at identifying molecules with a similar activity profile across phenotypic and macromolecular targets to that of a query molecule used as search template. VS using 3D similarity methods have the advantage of biasing this search toward active molecules with innovative chemical scaffolds, which are highly sought after in drug design to provide novel leads with improved properties over the query molecule (e.g. patentable, of lower toxicity or increased potency). Ultrafast Shape Recognition (USR) has demonstrated excellent performance in the discovery of molecules with previously-unknown phenotypic or target activity, with retrospective studies suggesting that its pharmacophoric extension (USRCAT) should obtain even better hit rates once it is used prospectively. Here we present USR-VS (http://usr.marseille.inserm.fr/), the first web server using these two validated ligand-based 3D methods for large-scale prospective VS. In about 2 s, 93.9 million 3D conformers, expanded from 23.1 million purchasable molecules, are screened and the 100 most similar molecules among them in terms of 3D shape and pharmacophoric properties are shown. USR-VS functionality also provides interactive visualization of the similarity of the query molecule against the hit molecules as well as vendor information to purchase selected hits in order to be experimentally tested.

## INTRODUCTION

Ultrafast Shape Recognition (1) (USR) is a molecular shape similarity technique enabling extremely rapid search for molecules with similar 3D shapes. In USR, each shape is encoded by a vector of 12 geometrical features derived from four strategically-located reference points with similar locations in similar molecules, thus providing a common framework for the comparison (2) (each point gives rise to a distribution of atomic distances, which is in turn characterized by its three first statistical moments acting as features). To date, USR has been successfully applied to a range of problems such as enabling real-time queries in integrative molecular databases (3–7); tabu search for protein conformations (8,9), atomic clusters (10) or protein-bound ligands (11); searching for non-peptidic inhibitors of protein-protein interactions (12); polypharmacology prediction (13) or enabling the development of other ultrafast methods based on the same principle (14–18).

This paper describes USR-VS, a user-friendly USR-based web server to perform large-scale ligand-based Virtual Screening (VS), which is oriented to the prospective validation of the obtained results. VS (19,20) aims at identifying previously-unknown active molecules for a target of interest and has become an area of wide interest owing to numerous cost-effective prospective applications (21–27). Ligand-based VS consists in searching for molecules similar to a query molecule known to be active against the target/s of interest. There is a plethora of molecular similarity techniques (28), although those based on molecular shape similarity are particularly suited for VS. Indeed, a certain degree of shape complementarity between a drug molecule and its intended therapeutic target is necessary for binding (29). Consequently, searching a molecular database for molecules that most closely resemble the shape of a given query molecule with known activity for a target is a fruitful strategy to identify other molecules with activity for that target. Furthermore, similar molecular shapes can be supported by strikingly different chemical scaffolds, which would lead to the valuable discovery of new chemical series for the target of interest. In this context, USR has discovered a high proportion of innovative inhibitors for molecular targets (falcipain-2 (30), arylamine NATs (31), DHQase2 (32), PAD4 (33), PRL-3 (34)) and phenotypic targets (colon cancer cell lines (35)). In addition to these prospective validations, USR has also been retrospectively validated for additional molecular targets (2,36).

A major extension of USR has been UFSRAT (37). UFSRAT searches for molecules that are not only similar in shape, but also similar in their spatial distribution of pharmacophoric features. This was achieved by segregating atoms into four overlapping categories (heavy, hydrophobic, hydrogen bond acceptor or donor atoms) and then calculating USR features from each set of atoms leading to the 48 UFSRAT features. In this way, UFSRAT screens for molecules that are likely to be complementary in shape with the implicit binding site and have a mechanism of binding similar to that of the query molecule. UFSRAT was successfully applied (34,38,39) to the discovery of novel inhibitors of four targets (PRL-3, MDM2, FKBP12 and HSD11B1). On the other hand, USRCAT (16) extended UFSRAT by adding a fifth category, aromatic atoms, intended to discriminate between long chain-like molecules such as some heteropeptides and long alkylchains. Furthermore, unlike UFSRAT, USRCAT employs the same four reference points, derived from all the heavy atoms of the molecule, to calculate the features for each of the five sets of atoms, which enhances virtual screening performance (16). As USR (2) and UFSRAT (38), USRCAT has demonstrated an excellent ability to retrieve active molecules with a different chemical scaffold than that of the query molecule, while sharing the common advantages of compact storage of features and extremely fast screening.

USR-VS is the first web server making USR and USRCAT available for large-scale prospective VS. This website is free and open to all users (there is no login requirement). There is only a similar web server (38) at http://opus.bch.ed.ac.uk/ufsrat/, which implements UFSRAT to screen a maximum of 4 853 000 conformers. However, the value of having alternative molecular similarity methods available is that these lead to the discovery of different active molecules for the same target (40). This is also the case even for methods based on the similar principle and screening the same set of molecules, as shown prospectively in this study (34) using freely-available and commercial shape similarity methods. In addition, USR-VS has several substantial advantages over the UFSRAT web server. First, USR-VS screens a much larger database comprising 93.9 million 3D conformers (23.1 million purchasable compounds). Second, these compounds come from ZINC database, which is not screened by UFSRAT (there are other web servers mining the ZINC database, but these employ different types of virtual screening methods such as 2D fingerprints (41) or docking (42)). Third, a highly optimized implementation of the alignment-free methods USR and USRCAT reaching formidable speeds of over 55 million 3D conformers per second, which permits providing the results in about 2 s. Lastly, unlike the UFSRAT web server, USR-VS provides interactive visualization and interpretation of the results via the hardware-accelerated WebGL visualizer iview (43), without using Flash or Java plugins.

## USR-VS WEB SERVER

The purpose of USR-VS is to provide a single resource where the user can bring a 3D conformer of a molecule with activity against macromolecular and/or phenotypic targets of interest and obtain a set of similar molecules ready to be purchased and experimentally validated on those targets. As the underlying ligand-based VS techniques have already been shown (2,16,30–36) to excel at identifying new active molecules with innovative chemical scaffolds for a range of targets, it is expected that a high proportion of USR-VS hits will have a similar profile of activity across targets than the query molecule.

The description of USR-VS can be broken down into the following areas: preparing the query molecule selected by the user as input data, the employed similarity techniques to identify the most similar database molecules to the query molecule, the construction of the screening database, running the virtual screen on this infrastructure and visualizing the results of the virtual screen. Furthermore, in addition to this high-level description, we will finish the section by outlining the highly optimized implementation of both 3D similarity methods in the web server.

### Input data from the user

An SDF file specifying a 3D conformer of the query molecule must be provided by the user to run USR-VS. There are several ways to obtain these files. The user can search for a given molecule, e.g. a marketed drug, in PubChem (44) and download the SDF with the modeled 3D conformer from this resource. To use the binding conformation of the query molecule, an SDF with a crystal pose of this molecule may be available for download at the Protein Data Bank (45). If this is not the case, this binding conformation can be predicted using user-friendly docking tools such as the idock web server (42). Another route is looking for active molecules for a given target in the ChEMBL database (46) and use software implementing a conformer generator, e.g. Frog2 (47), to obtain their 3D conformers from the downloaded chemical structures. To calculate the lowest energy 3D conformer of a compound, we have included in the help (http://usr.marseille.inserm.fr/help/) software similar to that used to generate the diverse 3D conformers screened by USR-VS.

### 3D molecular similarity

USRCAT extends shape-only USR by segregating the atoms of a 3D conformer into five overlapping categories (heavy, hydrophobic, aromatic, hydrogen bond acceptor or donor atoms) and calculating USR features for each of the resulting sets of atoms. Consequently, the number of features per conformer is expanded from 12 to 60, with the first 12 features being identical in both methods.

### Screening database

A USR-VS run screens 93 903 333 low-energy 3D conformers generated from 23 129 049 purchasable compounds collected from the All Clean subset of the ZINC database (48). The very large size and diversity of this screening database maximizes the likelihood of containing similar molecules to any given query molecule. We used a recent protocol for conformer generation based on RDKit (http://www.rdkit.org/), which was found to offer the best results in accuracy and second best in terms of efficiency (49). This includes a post-processing algorithm to discriminate and keep only those conformers of a given molecule that are both energy-minimized and conformationally diverse. As a result, an average of four conformers per molecule were generated, with only one conformer retained for more than six million molecules and a maximum of 35 conformers generated for some molecules.

### Running a virtual screening

This is performed in three simple steps. The first is to upload the SDF file containing the 3D conformer of the selected query molecule. Second, the user must select either USR or USRCAT as the similarity method providing the score with which to rank database molecules (as customary, the score between two compared molecules is defined as the highest score between conformers from each molecule). We recommend prospectively testing hits from both methods, as this typically leads to the discovery of different active molecules for the same target, e.g. (34). Third, clicking the submit button to launch the virtual screen with these choices, which will start as soon as any previous screens from other users have been completed.

### Inspecting the results

Once the virtual screen is completed, the user is redirected to the result webpage with a unique URL. This URL is only available to the user, who is recommended to place a bookmark on it if they wish to access the results at a later time (the result URL will be kept for at least two years). At the bottom left of the page, there are two output files (hits.csv and hits.sdf) for downloading information about the top 100 most similar molecules to the query molecule. The hits.csv file provides compound IDs, similarity scores (USR, USRCAT and Tanimoto score based on 2D Morgan fingerprints), physicochemical properties, SMILES and vendors selling the hits. The latter does not contain the vendor IDs for the hits, which can be found by following the vendors and annotations link on the right of the result webpage. The annotations might include known targets for the hit compound, which are also to investigate whether the target of interest has already been tested for that compound. The hits.sdf file contains the 3D conformers of the hits in SDF format. Please note that, although USR and USRCAT scores are provided for these top hits, the identity of the hits depends of the user-selected method used to rank them, either USR or USRCAT.

**Figure 1.**
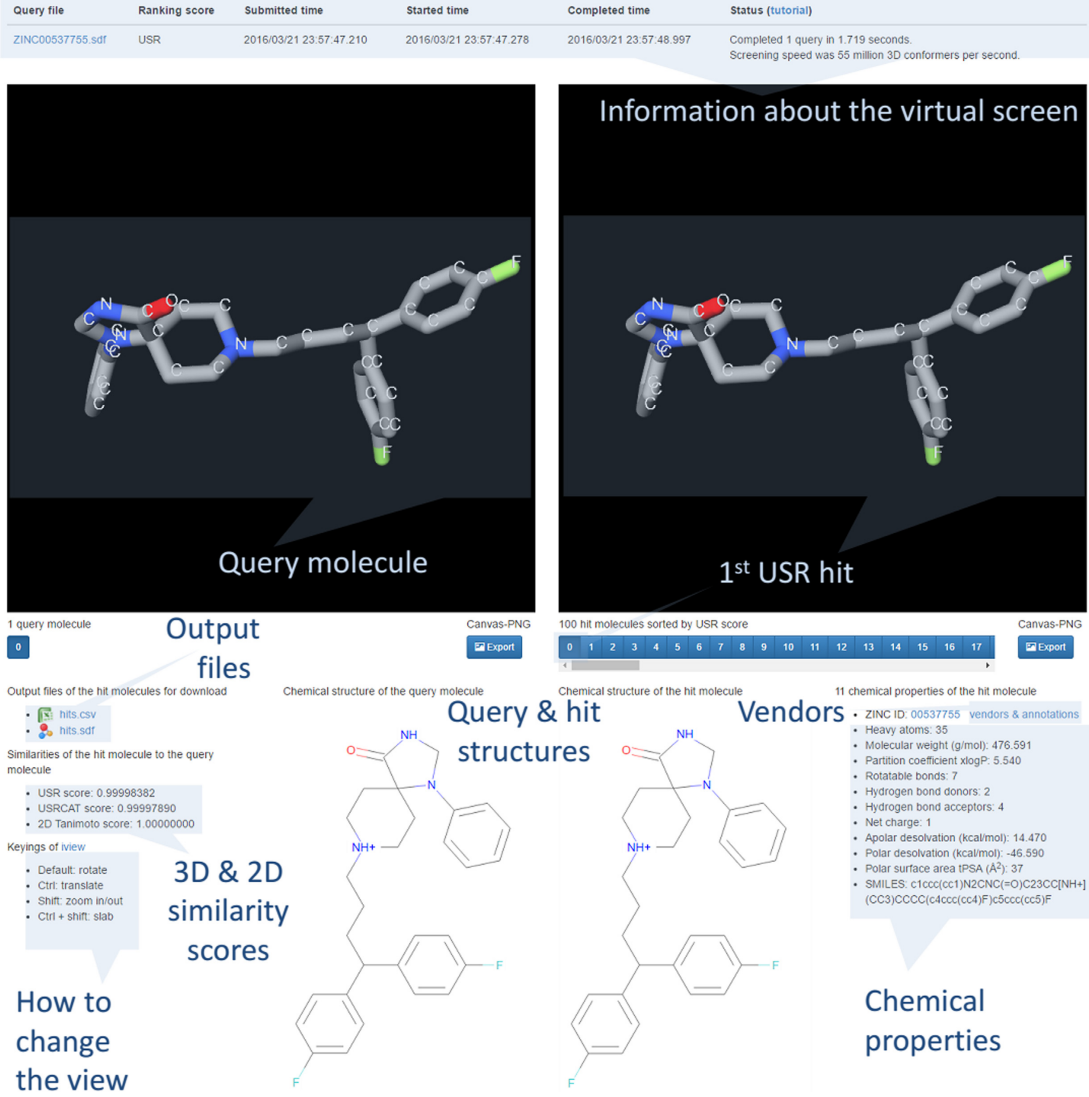
USR-VS results webpage for fluspirilene.

As shown in Figure 1, the top hits are also visualized in a hardware-accelerated 3D WebGL canvas using iview (43) without using Flash or Java plugins. The left canvas shows the 3D conformer of the query molecule, whereas the right canvas presents that of the inspected hit. The user can switch among the top 100 hit molecules by pressing the buttons below the right canvas (these are sorted from 0 to 99 by decreasing similarity score with the query molecule) and inspect the corresponding 3D and 2D similarity scores, chemical properties and several options for purchasing the compound. These hits, but not all database molecules, were aligned with the query molecule by calculating the 3D rotation that superposes the four reference points of each molecule. In addition, any hit molecule can be interactively translated, rotated and zoomed in/out to change the relative orientation of the compared molecules by dragging and scrolling with the mouse (selected orientations of any molecule can be exported as a PNG image). This permits an appreciation of the degree of 3D similarity of the compared molecules, which is intended to help the user decide which hits to purchase and how to purchase them to experimentally measure their activity against selected targets of the query molecule. In addition, the chemical structures of the compared molecules are also shown in order to identify cases of chemical scaffold hopping among the hits.

### Implementation

USR-VS is currently able to screen over 55 million 3D conformers per second. Thus, this web server only takes about two seconds to calculate 3D similarity scores of 93.9 million conformers, ranking 23.1 million molecules and writing results to file for the top 100 most similar molecules for the consideration of the user.

This unusual level of efficiency is mainly due to the following factors. First, the alignment-free and highly compact encoding of 3D molecular properties achieved by USR and USRCAT. Second, all USRCAT features for the entire set of conformers (42 GB in size) are preloaded into memory during the web server startup. This is a one-off exercise, which avoids loading these features for every query at the cost of constantly occupying 42 GB of memory on the server side (this is estimated to save about 100 s per query using SSDs). Third, USR-VS employs a multithreading design running on just *n* = 12 CPU cores, with each core screening an average of *P* = 16 database molecule chunks of size N/(p*n), where *N* = 23 129 049 compounds and p was set as the lowest parameter value ensuring a dynamic balance load across the cores (unbalances are due to uneven number of conformers between chunks and uneven share of CPU time among threads). Lastly, a heuristic to reduce the time to process conformers while comparing two molecules. In this case, we compare the conformer of the query molecule with the c conformers of the database molecule (c ranges from 1 to 35). Each of these c dissimilarity scores takes the form of a sum of absolute differences (SAD) between the two 60-dimensional vectors. Since only the conformer with the minimum SAD is required (i.e. that with the highest similarity score), a short circuit mechanism is implemented whereby the SAD calculation is halted as soon as the current minimum SAD is exceeded. This saves the time of calculating the remaining feature differences between the query and that conformer and it is particularly important for those molecules where the conformer with the highest similarity score happens to be processed early.

## EXAMPLES

### Example 1: Fluspirilene

Fluspirilene is an antipsychotic drug contained in our screening database. Figure 1 shows the results of using USR to search for similarly shaped molecules to fluspirilene. This query molecule is shown on the left canvas, whereas information about the completed virtual screen is displayed in a table above. Hits can be interactively visualized with iview. On the right canvas, the top 100 USR hits from the virtual screen can be displayed by pressing the corresponding numbered button below (e.g. number 6 for the seventh most similar USR hit). The first USR hit is fluspirilene itself as indicated by a Tanimoto score of 1, which is one of the 23.1 million molecules in the screening database. The view of the displayed molecules can be changed with the keyings located at the bottom left. Above this, two output files with all the information about the hits can be downloaded. The bottom middle of the results page show the chemical structures of the query and selected hit. The bottom right displays the chemical properties and vendors&annotations link for the currently visualized hit, respectively (in this case, the link also leads to cross-references to additional information, as fluspirilene is a marketed drug).

### Example 2: Vemurafenib

**Figure 2.**
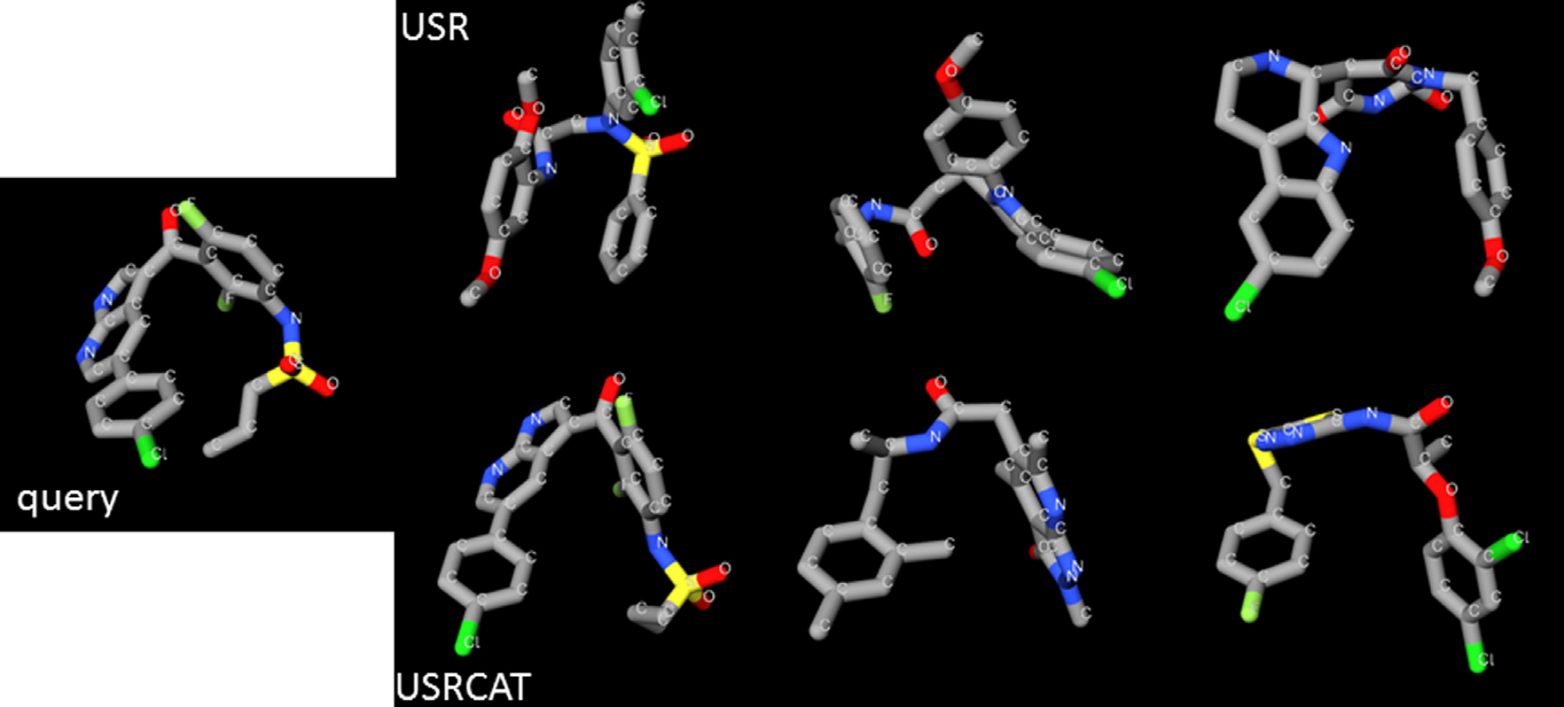
USR-VS hits for vemurafenib.

Figure 2 shows the results for PubChem's 3D conformer of the cancer drug vemurafenib shown on the left (https://pubchem.ncbi.nlm.nih.gov/compound/42611257#section=3D-Conformer). On the right, the top row shows the three top hits according to USR, whereas the bottom row shows the three top hits according to USRCAT (the closer to the left, the higher the similarity score). Note that similar hits are obtained by both methods, with some similarly shaped molecules representing large chemical scaffold hops from the query molecule (e.g. the third hit from each method).

## CONCLUSION

USR and USRCAT have exhibited an excellent ability to identify active molecules with innovative chemical structure in previous studies. However, many users without programming and chemical informatics knowledge, but with the capacity to perform wet-lab assays to validate the predicted drug-target associations, are discouraged by technical barriers. Here we have presented USR-VS, a highly optimized user-friendly web server for prospective virtual screening powered by these methods. We hope that USR-VS will contribute to making virtual screening more widely used in experimental labs.
